# Wounding the stroma: Docetaxel’s role in dormant breast cancer escape

**DOI:** 10.1371/journal.pbio.3002297

**Published:** 2023-09-13

**Authors:** Tyler T. Cooper, Lynne-Marie Postovit

**Affiliations:** Biomedical and Molecular Sciences, Queen’s University, Kingston, Canada

## Abstract

The mechanistic underpinnings of breast cancer recurrence following periods of dormancy are largely undetermined. This Primer explores a new study in PLOS Biology which reveals that docetaxel-induced injury of tumor stromal cells stimulates the release of cytokines that support dormancy escape of breast cancer cells.

## Teaser

The mechanistic underpinnings of breast cancer recurrence following periods of dormancy are largely undetermined. A new study in *PLOS Biology* reveals that docetaxel-induced injury of tumour stromal cells stimulates the release of cytokines that support dormancy escape of breast cancer cells.

Elucidating cellular mechanisms that underlie breast cancer recurrence following surgery, chemotherapeutics, and chronic inflammation is critical to improving therapeutic efficacy [1]. Despite the growing recognition of the tumour microenvironment’s (TME) role in awakening dormant cancer cells, details of cellular communication networks remain largely unresolved [2]. In a recent report in *PLOS Biology*, Ganesan and colleagues illuminate a hitherto unknown mechanism of breast cancer recurrence wherein the TME drives progression [3]. The investigators provided evidence that administration of docetaxel can modify the stromal cell secretome and awaken dormant cancer cells by promoting an immunosuppressive and pro-metastatic TME conducive to cancer recurrence. Notably, the authors identified MEK/ERK as a targetable signalling hub to prevent the “awakening” of dormant breast cancer cells. These findings advance our understanding of breast cancer dormancy and could inform future therapeutic strategies aimed at preventing cancer recurrence.

To track dormancy escape, the authors employed a fluorescence ubiquitin cell cycle indicator (FUCCI) reporter system [4] within 3D breast cancer spheroids in monotypic culture or in combination with stromal and endothelial cells, termed tumour stromal organoids (TSOs). The FUCCI reporter system is comprised of 2 fluorescent reporter constructs that allow for the monitoring of cell cycle arrest through expression of a red fluorescence reporter (mKO2-cdt1) as well as reentry into the cell cycle through the transitional expression of a green reporter fluorescence construct (Clover-Gremlin). Additionally, investigators leveraged single-cell RNA sequencing (scRNA-seq) with TSO cultured in reduced growth factor basement membrane extract (RGF BME) to show that TSO maintained a dormant state and that docetaxel activated cell cycle pathways in cancer cells while decreasing stromal cell proliferation. Notably, cancer cells cultured together with stromal cells (but not as monoculture) could overcome dormancy following drug treatment. Cancer cells from docetaxel-treated tumour stromal culture also exhibited an increased migratory capacity through Matrigel and were better able to form lung metastases, suggesting that extrinsic stimuli within the TME promotes cancer cell awakening [5].

**Fig 1:**
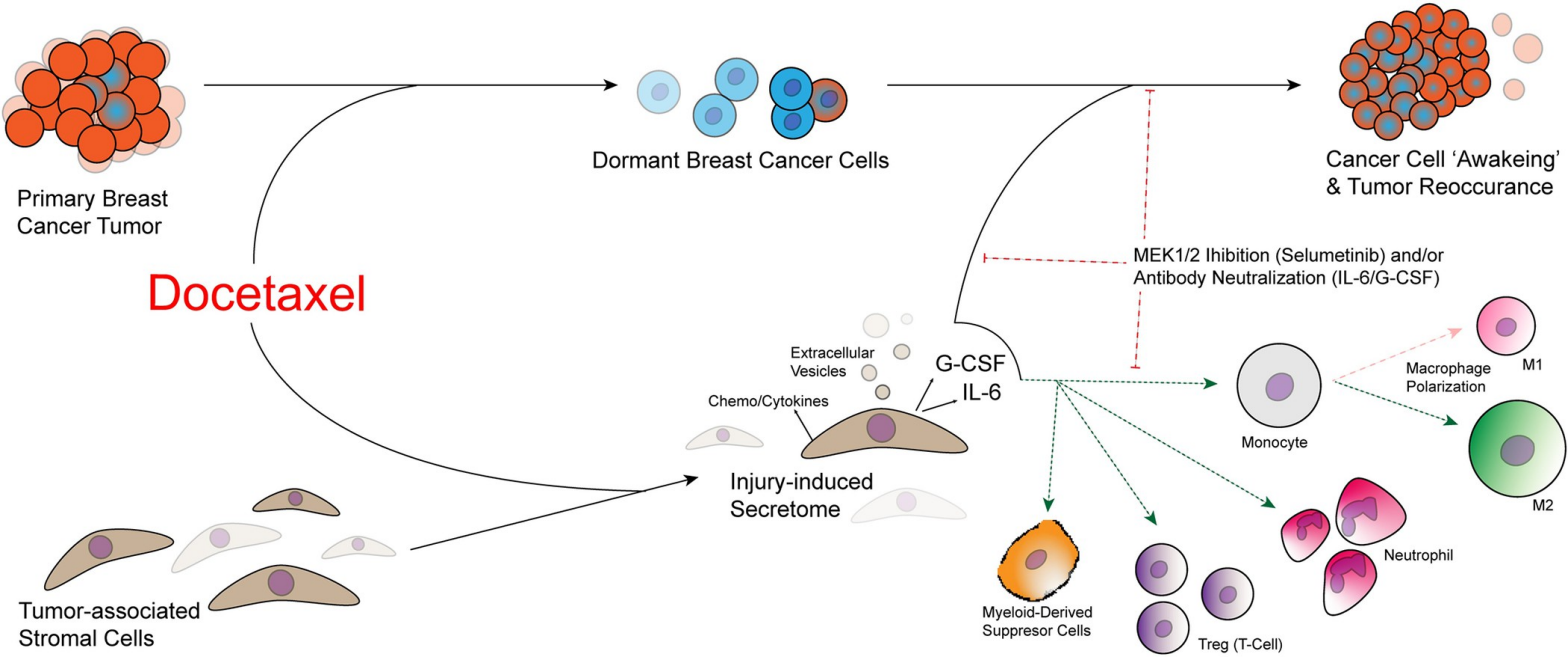
Docetaxel modulates the secretome of breast cancer-associated stromal cells that “awakens” dormant tumour cells and promotes an immunosuppressive microenvironment.

Hallmarks of cellular dormancy include a quiescent phenotype maintained within G0 cycle cell arrest [1] and a senescence-associated secretory phenotype (SASP) that paradoxically triggers cancer cell awakening [6]. To determine the extent to which SASP-associated factors were produced in response to therapy, the TSO secretome was examined by a 32-plex cytokine array that included chemo/cytokines such as IL-6, G-CSF, TNFα, and VEGF. The source of these factors was established with scRNA-seq and it was concluded that docetaxel-induced cancer cell proliferation is associated with IL-6 and C-GSF secretion from inflamed stromal cells (Fig 1). Neutralisation of IL-6/G-CSF was able to prevent docetaxel-induced activation of dormant cancer cells in vivo; however, it remains to be determined if IL-6/G-CSF inhibition can revert awakened cancer cells to a dormant state. This is an important caveat, as entry into dormancy is crucial for the adaptation of cancer cells to hypoxic and immune intolerant microenvironments.

scRNA-seq identified over 1,500 differentially regulated genes following a single dose of docetaxel, many of which were linked to chemoresistance, tumourigenesis, and increased cancer invasiveness. Several cancer-related pathways were activated, with a striking up-regulation of ERK-related genes. These findings support previous reports of an increased ERK/p38 ratio in metastatic cancer [7]. Conditioned media from docetaxel-treated stromal cells increased ERK-activation in TSOs and selumetinib, an MEK1/2 inhibitor, prevented chemotherapy-induced dormancy escape in vitro and in vivo, reducing cancer cell proliferation without impacting stromal cells. This suggests that factors secreted from stromal cells in response to therapy cause dormancy escape, at least in part by activating ERK signalling in cancer cells. Likely candidates for this activation are IL-6 and G-CSF as neutralisation of IL6/G-CSF/MEK1/2 mitigated phosphorylation of ERK concomitant with reductions in cancer cell proliferation and invasion.

To further probe the TME, investigators utilised flow cytometry and scRNA-seq to study the immune cell landscape in dissociated tumours. Tissue resident immune cells, such as macrophages and neutrophils, have an established role in maintaining cancer cell dormancy while paradoxically promoting “awakening” upon activation [4,8]. A shift towards a pro-tumour landscape was induced following docetaxel treatment. Specifically, an increase of myeloid-derived suppressor cells, neutrophils, and M2 macrophages, was observed within the TME (Fig 1). Immunosuppressive regulatory T cell populations were also increased, coinciding with a diminished anti-tumour CD8+ T cell population independent of CD4+ cell proliferation. Inhibition of IL-6/G-CSF/MEK1/2 shifted the immune cell landscape towards an anti-tumour profile, suggesting that these pathways also instruct a pro-tumourigenic immune TME.

Collectively, this study underscores the unintended consequence of chemotherapy and suggests MEK/ERK signalling as a potential therapeutic target to prevent the awakening of dormant cancer cells. While inducing awakening from dormancy may promote metastatic potential and recurrence, it may also be exploited to make cancer cells more sensitive to chemotherapeutics. Thus future studies should compare and contrast strategies that directly target dormant cells to those wherein exit from dormancy is prevented to maintain a non-metastatic state [1]. This study did not consider the mechanisms by which docetaxel affected the stromal cells. However, taxanes arrest active cells in G2/M through binding of beta-tubulin and reducing anti-apoptotic BCL2 levels [9]. Hence, it is likely that docetaxel directly perturbs stromal and immune cell phenotypes.

The TME drives cancer progression by promoting cancer stem cell self-renewal, plasticity, chemoresistance, and immunotolerance through autocrine and paracrine signalling networks [2,4,5,8]. Cellular (stromal, endothelial, immune cells) and noncellular components (extracellular vesicles, cytokines), along with extracellular matrix composition influence transitions into a dormant versus awakened cancer cell state. Additional research has emphasised the role of hypoxia underlying dormancy and chemoresistance by promoting a stem cell phenotype in cancer cells through parallel modifications to translational, metabolic, phenotypic, and secretory networks [5]. Although not explored, the mTOR signalling network was highly modified in docetaxel-treated TSO and can regulate both translational and metabolic machinery in cancer cells to regulate a cancer cell dormancy [10]. Nucleic acid metabolism or fatty acid oxidation networks may also regulate dormancy [10] and metastasis.

The extent to which these processes are regulated by stromal-derived secretome inside and outside the TME are yet to be fully understood. Wounding of stroma outside of the TME has been shown to augment secondary tumour formation in immunocompetent mice with mammary fat pad tumours [11] and inflammatory treatments in tumour-resection models reduced secondary tumour formation [12]. Systemic IL-6 and G-CSF levels are elevated during surgical wounding and chronic inflammation; thus, it would be interesting to contrast the inflammatory secretome produced following chemotherapy versus surgical wounding encountered during tumour resections. Do other pro-inflammatory cytokines directly awaken breast cancer or do stromal cells act as both recipient and messenger? Are the mechanisms of inflammation-associated cytokines concentration and/or cell specific? While the current study illuminates a mechanism by which the TME may promote an exit from dormancy, further studies are needed to consider additional dynamic reciprocities that may promote cancer progression.

## Abbreviations

FUCCI: fluorescence ubiquitin cell cycle indicator
RGF BME: reduced growth factor basement membrane extract
SASP: senescence-associated secretory phenotype
scRNA: seq, single-cell RNA sequencing
TME: tumour microenvironment
TSO: tumour stromal organoid
